# Early childhood severe scalds in a developing country: A 3-year retrospective study

**DOI:** 10.4103/2321-3868.123073

**Published:** 2013-12-18

**Authors:** Pius Agbenorku

**Affiliations:** 1 Reconstructive Plastic Surgery and Burns Unit, Department of Surgery, Komfo Anokye Teaching Hospital, School of Medical Sciences, College of Health Sciences, Kwame Nkrumah University of Science and Technology, Kumasi, Ghana; 2 Reconstructive Plastic Surgery and Burns Unit, Komfo Anokye Teaching Hospital, School of Medical Sciences, College of Health Sciences, Kwame Nkrumah University of Science and Technology, Kumasi, Ghana

**Keywords:** Scalds, risk factor, education, early childhood, outcome, prevention

## Abstract

The burns intensive care unit (BICU) staff observed an increasing number of pediatric scald burn admissions as a result of increase injuries associated with the scald burns. A retrospective study was conducted to identify scalds demographics, etiologies, and mortality risk factors. This descriptive study comprised a total of 166 patients aged 0-5 years, who were admitted to the BICU of the Reconstructive Plastic Surgery and Burns Unit (RPSBU) through the Accident and Emergency (A and E) Centre of the Komfo Anokye Teaching Hospital (KATH)from May 1^st^ 2009 to April 30^th^ 2012. Source of information was the BICU Computerized Database System. Data extracted included demographics as well as treatment methods and outcomes. The study population was 166; 92 (55.4%) males and 74 (44.6%) females. Scalds admissions were 141 (84.9%); 13 (9.2%) of them died, 83 (58.9%) discharged, and 45 (31.9%) transferred-out to another burn ward and pediatric surgery ward in the hospital. Scald patients’ demographics included 78 males (55.3%) and 63 females (44.7%); mean age was 2.18 years. Mortality risk factors identified were age <3 years (P = 0.044); scalds from hot water (P = 0.033), total burns surface area >30% (*P* = 0.017), and multiple body parts affected (*P* = 0.049). The current study showed age, hot water, and Total Burns Surface Area (TBSA) as risk factors of early childhood scalds. Education on scalds prevention targeting mothers/caregivers is needed to create awareness of the frequency, severity, and danger associated with pediatric scalds.

## Introduction

Globally, scalds are the most commonly treated burns that generally result in less severe injuries and rarely require a lengthy hospital stay[1,17] Scalds may be superficial, deep, or a combination of both. Among children under 6 years, falls, poisoning, cuts, burns, smoke inhalation, drowning, suffocation, and choking cause at least two-thirds of all unintentional injuries. Many of these injuries occur in the home where young children spend most of their time;[18] thus they are often scalds.

**Table:** 

Access this article online	
**Quick Response Code:**	**Website:** www.burnstrauma.com
	**DOI:** 10.4103/2321-3868.123073

Burns from hot drinks, food, steam, or other hot liquids (all referred to as ‘scalds’) are common causes of serious injuries to young children. According to the booklet, *“A Child Safe Home for Under Fives: preventing injuries to young children”*,[19] especially under-fives are naturally curious and are often attracted by the steam from hot water or drinks. They may not recognize the danger of getting burnt until it is too late. While there are enormous variations in the age of patients with burn injuries, global studies demonstrate that the 0–5 year age group had the highest incidence.[1–3,7–9,16,20–25] The high incidence of burn injuries in this age group is attributable to children’s impulsiveness, lack of awareness, higher activity levels, natural curiosity, and total dependency on their caregivers.[26–28] In toddlers, the upper part of the body including the anterior trunk is the most frequently burned area.[1,26,29] Burns of the upper body are associated with the natural curiosity of the 0–5 year age group exploring their surroundings through pulling, touching, and grabbing objects.[1] Burns mortality factors such as gender, age, comorbid disease, inhalation injury, co-existing trauma, and pneumonia have been identified and reported.[30,31] This study seeks to identify early severe scalds demographics, etiologies, and mortality risk factors in children aged 0–5 years.

## Materials and methods

### Study setting

The Komfo Anokye Teaching Hospital (KATH) in Kumasi is the second largest hospital in Ghana and the only tertiary health institution in the middle belt of the country. It is the main referral hospital for the Ashanti, Brong Ahafo (BA), northern, upper east, and upper west regions. Statistical records from KATH show that about two-thirds of the patients are from the Ashanti Region, with BA and the three Northern regions sharing the remaining in a two to one ratio. The hospital was built in 1954 as the Kumasi Central Hospital. It was later named Komfo Anokye Hospital after Okomfo Anokye, a legendary fetish priest of the Ashantis. It was converted into a teaching hospital in 1975, affiliated to the School of Medical Sciences of the Kwame Nkrumah University of Science and Technology (KNUST) in Kumasi. The hospital currently has 1,000 beds, up from the initial 500 when first built. Annually, the hospital attends to about 679,050 patients made up of both out- and inpatients (KATH Biostatistics Unit, 2012).

In order to assess the causes and magnitude of scalds children 0–5 years of age with scalds wounds that were admitted to KATH burns intensive care unit (BICU) were eligible for the study. Consent was sought and obtained from the parents/guardians/caretakers of the affected children whose data was used in the study.

### Ethical clearance

Ethical clearance for this study was obtained from the KNUST School of Medical Sciences/KATH Committee on Human Research, Publication and Ethics, Kumasi.

### Data collection

Data on the patients were collected from May 1^st^ 2009 to April 30^th^ 2012 from the Computerized Database System at the BICU of the Reconstructive Plastic Surgery and Burns Unit (RPSBU). Data retrieved for the study included: Admissions, demographics, extent of burns, etiology of burns, length of hospital stay, and mortality.

### Data analysis

The quantitative data was presented using descriptive statistics, summarized, and displayed on graphs and charts. Data entry and analysis was made using Statistical Package for Social Sciences (SPSS) version 17.0 (SPSS, Inc., Chicago, IL, USA).

### Patient management

Burn injuries in all age groups were admitted first into the Accident and Emergency (A and E) Centre following the triage process and subsequent initial admissions to its various sections: First Aid Unit — *Yellow Section*; Minor Treatment Unit — *Orange Section*, and Major Treatment Unit —*Red Section* were done according to the burn injury severity as prescribed in the KATH A and E Centre Burn Protocol.[32] This same Protocol was strictly followed for the admissions and immediate treatment/resuscitation. Similarly, further management procedures were done according to the same Protocol.

### Limitations of the study

This study does not cover all the scald injuries in KATH since some of them, being minor, were treated on outpatient basis, hence their records were not captured in the BICU.

## Results

### Sex distribution

**Figure 1:**
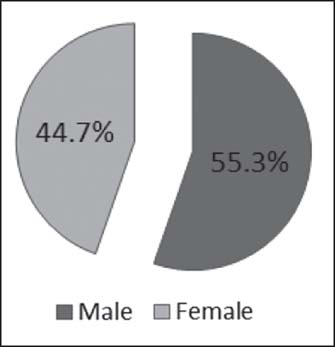
Sex distribution of scalds in age group 0–5 years (*n* = 141).

Scald burn patients’ demographics included males 78 (55.3%) and females 63 (44.7%) with a mean age of 2.18 years [Figure 1].

### Etiology of burns

**Figure 2:**
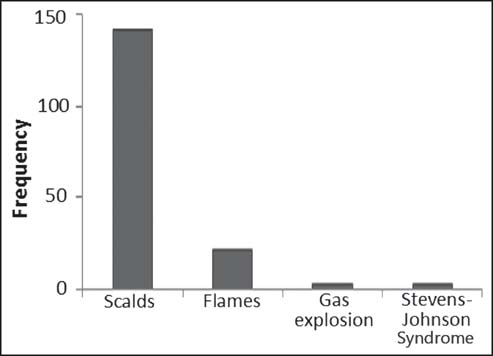
Etiology of burns in age group 0–5 years (*n* = 166).

The mean total body surface area (TBSA) was 19.7% with mean length of BICU admission of 6.03 days. The remaining pediatric burns admissions (N = 166 – 141 = 25) for children aged 0–5 were caused by open fire or flame (21, 12.7%), gas explosion (2, 1.2%), and Stevens-Johnson Syndrome (2, 1.2%) [Figure 2].

### Etiology of scalds

**Figure 3:**
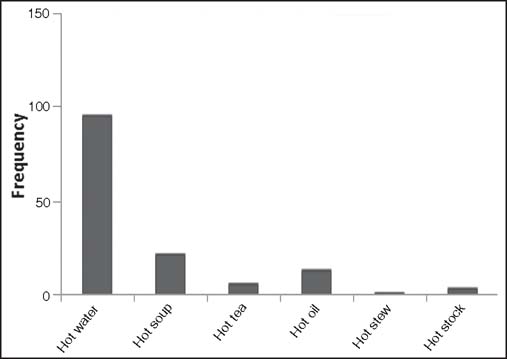
Etiology of scalds (*n* = 141).

**Figure 5:**
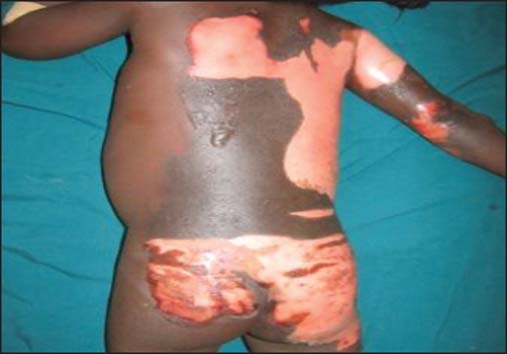
Early childhood severe scald caused by hot soup.

**Figure 6:**
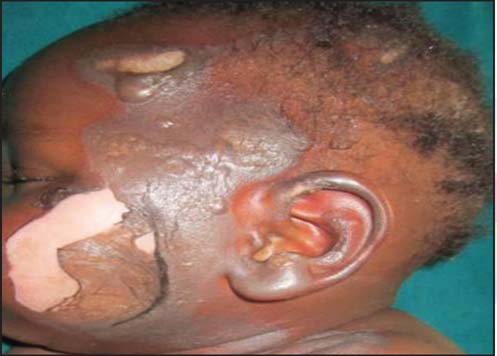
Early childhood severe scald caused by hot tea.

**Figure 7:**
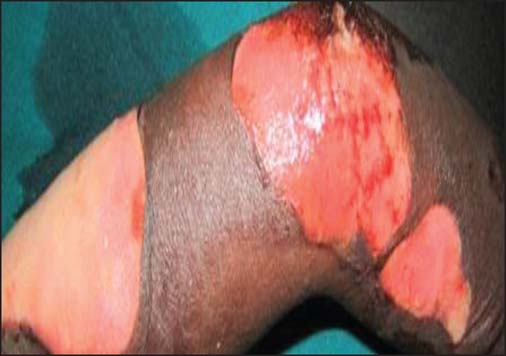
Early childhood severe scald caused by porridge.

The etiological agents for the scald burns included: Hot water (96, 68.1%)—the commonest, hot soup (22, 15.6%), hot tea or porridge (6, 4.3%), hot oil (13, 9.2%), hot stew (1, 0.7%), and hot stock (3, 2.1%) [Figures 3, 5, 6 and 7].

### Outcome of scalds

**Figure 4:**
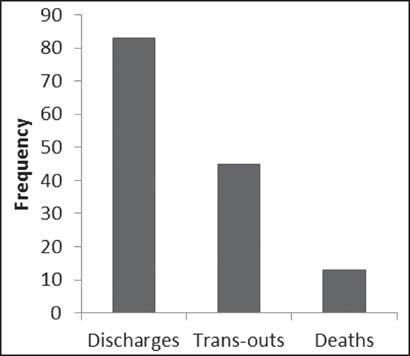
Outcome of scalds (*n* = 141).

Out of the 141 scald burns there were 13 (9.2%) deaths, 83 (58.9%) discharges, and 45 (31.9%) transferred-outs to other burn wards in the hospital (General Burns Ward and Pediatric Surgical Ward) [Figure 4].

### Risk factors that influenced burn mortality in the study

Multiple regression analysis was used to determine sociodemographic features which are mortality predisposing factors influencing pediatric burns in the study center. Using the “outcome of admission, that is, death” as the dependent factors, all the demographic features of the participants were involved in the analysis. A probability value (*P*-value) of less than 0.05 was considered to be statistically significant at 95% confidence interval [Table 1].

**Table 1:** Pediatric burns mortality risk factors in the study

	Unstandardized coefficients		Standardized coefficients		
Model	B	Standard error	Beta	*t*	Significance (*P*-value)
Age (<3 years)	0.097	0.022	0.620	1.235	0.044**
Sex	0.140	0.064	0.301	1.064	0.063
Duration	−0.032	0.038	−0.364	2.347	0.451
Etiology (hot water)	−0.056	0.011	−0.244	1.082	0.033**
TBSA (>30%)	−0.077	0.063	−0.295	−2.111	0.017**
Inhalation injury	0.084	0.037	−0.187	1.065	0.074
Body part affected (multiple parts)	0.107	0.143	2.034	0.710	0.049*

**Denotes significant value (*P*< 0.05.TBSA = Total body surface area

Thus in this current study, children less than 3 years, hot water, TBSA greater than 30%, and multiple body parts affected were identified as mortality risk factors of scalds in children aged 0–5 years.

## Discussion

The psychological stress burns patients go through could have crippling effects on them such as causing them to be nonfunctional and thereby affecting their quality of life.[33] Due to the curious nature of infants and toddlers and the urge to mostly pull and push things around, they end up getting injured when containers, pots, or pans containing hot fluids overturn and pour on them.[6] Most parents especially mothers and caregivers usually have a tough time handling children during this stage of their developments.

Infection following burn injuries is possible especially in children if not properly handled. Children who suffer from burns in general need special care and attention to help hasten the healing process as well as to minimize scarring. Scarring from scald injury could affect the individual psychologically.[33] From the current study, out of the 141 children who suffered scalds, there were 13 deaths (mortality = 9.2%), 83 discharges, and 45 transferred-outs to other burn wards. Outcome of scalds being death could be attributed in delay of taking the victim to the hospital or a specialized center where they can be properly taken care of; no death was recorded out of the patients transferred out or discharged. In their study on the characteristics of burns in children under 7 years of age, Balseven-Odabasi reported that 69% of burn injured children were less than 3-years-old and that scalds accounted for over 60% of pediatric burns while the mortality rate was 4%.[1] If victims are well taken care of some complications could be avoided. Scald injury could be extensive, affecting larger body surface area with deeper tissue injury than thermal burns resulting in hospitalization and transfer as occurred in about 25% of patients, whereas as high as 99.9% of thermal burns patients were treated and discharged.[6]

According to Cronin *et at.*, the majority of parents become aware of that these accidents are preventable after they had occurred.[34] Due to the fact that cognition in children is not as high as in adult, during their moving about physically, they are unaware of the associated risks involved hence increasing their ability of encountering hot fluids, thus as their motor skill development exceeds their cognitive development. Results from this study showed most of the scald injuries occurred in the kitchen (85%). Similar findings were reported by Serour *et al.*,[35] and Schricke *et at.*,[36] in their studies stating that scald injuries occurring in the homes were 85.7 and 90.8%, respectively and therefore in the homes, children or toddlers should not be allowed in the kitchen area unattended to.[36]

Even though strides have been made in developed countries to aid in the prevention of burns, the situation is however different in developing countries and low middle income countries. In the current study, the major factor that accounted for increased early childhood scalds admission was lack of supervision by mothers and caregivers. Caregivers and parents should be offered training to properly take care of their children. Public education at all levels on burns should be encouraged. The elderly should keep kids under very close supervision or surveillance. Behaviors in the home can also result in increased scald occurrence such as placing hot drinks at places where toddlers can easily have access to can increase the risk.

A study by Serour *et al.*, also showed that males (69.2%) outnumbered females (30.8%) in their 5-year study period on 266 children who were hospitalized for thermal burn injury.[35] Our current study also shows that males (55.3%) suffered more from scalds than females (44.7%). In the current study, hot water (68.1%) was the major etiology of scalds; this finding is similar to the study by Balseven-Odabasi *et al.*, where they reported hot water (81.7%) as the major cause of scald injury.[1] However, in a retrospective study carried out by Yates *et al.*, hot beverage accounted for 65% of all scalds, followed by hot water (16%), and then hot foods such as soup and sauces (10%).[32] Hot water was the scald agent in 48.5% cases reported by Drago in her study where she also revealed that scalds contributed 65.7% of burns treated injury during a 6-year period of 17,237 emergency department treated burns in children 5 years and younger.[6]

In this study, scalds (84.9%) were the major etiology of burns which was similar to what Balseven-Odabasi *et al.*, reported that scalds accounted for 82% of all injuries in their study.[1] Also, Serour *et al.*, reported in their study that scalds (74.4%) was the most common etiology of burns in their study[35] and similarly, Schricke *et al.*, reported that scald injury contributed 56.0% of all pediatric burns admissions in their study.[35] Increase in hospital stay results in high economic burden for families due to extra time and cost for persons managing victims of scalds and burns leading to economic loss and morbidity.[37,38]

Agbenorku *et al.*, in their study identified the following as some of the pediatric burns mortality risk factors: Persons below the age of 6 years; hot water or soup; and total burn surface area greater than 36%.[39] Similarly, in this current study; children less than 3 years, hot water, TBSA greater than 30%, and multiple body parts affected were identified as mortality risk factors of scalds in children aged 0–5 years.

Burns in general, irrespective of the etiology poses some socioeconomic burdens on the victims as well as caregivers resulting in morbidity and nonproductivity.[37,38] In general, infants and toddlers depend solely on their mothers and caregivers for care and support they need, implying that caregivers have to invest time which could otherwise be used to work to take care of their kids hence causing economic burden. This current study has as high as 84.9% being scald injuries and so burns preventive measures, especially towards scald prevention at homes should be paramount since the home serves as the highest place of childhood scalds occurrence.[33] Public education on scald injury prevention should be encouraged at all levels especially in schools where young children and their mothers/caregivers are likely to benefit from and also training programs on infant care and management should be promoted so that children are better taken care of always especially at homes to prevent occurrences of unfortunate incidences.[40]

## Conclusion

Age less than 3 years, scalds from hot water, TBSA greater than 30%, and multiple body parts affected were early childhood scalds mortality risk factors identified. Since scald injuries in children are very common, mothers and caregivers should always place precautionary measures when handling their infants and toddlers. Public education on safety and burns prevention should be encouraged at all levels. Mothers and caregivers should ensure children are not allowed unattended to in kitchen areas or places where they could be exposed to danger. In situations where burns occur, caregivers should take the necessary action by taking the child to a health facility so that the child gets care and appropriate treatment to avoid fatalities.
